# AFP promotes HCC progression by suppressing the HuR-mediated Fas/FADD apoptotic pathway

**DOI:** 10.1038/s41419-020-03030-7

**Published:** 2020-10-02

**Authors:** Tianke Chen, Xiaowei Dai, Juji Dai, Chaodong Ding, Zheng Zhang, Ziqi Lin, Jin Hu, Mei Lu, Zhanyu Wang, Yalei Qi, Li Zhang, Rulu Pan, Zhu Zhao, Liting Lu, Wanqin Liao, Xincheng Lu

**Affiliations:** School of Basic Medical Sciences, Wenzhou Medical University, 325035 Wenzhou, China

**Keywords:** Liver cancer, Oncogenes

## Abstract

Hepatocellular carcinoma (HCC) is a major leading cause of cancer-related death worldwide. Alpha fetoprotein (AFP) is reactivated in a majority of hepatocellular carcinoma (HCC) and associated with poor patient outcomes. Although increasing evidence has shown that AFP can regulate HCC cell growth, the precise functions of AFP in hepatocarcinogenesis and the associated underlying mechanism remain incompletely understood. In this study, we demostrated that depleting AFP significantly suppressed diethylnitrosamine (DEN)-induced liver tumor progression in an AFP gene-deficient mouse model. Similarly, knocking down AFP expression inhibited human HCC cell proliferation and tumor growth by inducing apoptosis. AFP expression level was inversely associated with the apoptotic rate in mouse and human HCC specimens. Investigation of potential cross-talk between AFP and apoptotic signaling revealed that AFP exerted its growth-promoting effect by suppressing the Fas/FADD-mediated extrinsic apoptotic pathway. Mechanistically, AFP bound to the RNA-binding protein HuR, increasing the accumulation of HuR in the cytoplasm and subsequent inhibition of Fas mRNA translation. In addition, we found that inhibiting AFP enhanced the cytotoxicity of therapeutics to AFP-positive HCC cells by activating HuR-mediated Fas/FADD apoptotic signaling. Conclusion: Our study defined the pro-oncogenic role of AFP in HCC progression and uncovered a novel antiapoptotic mechanism connecting AFP to HuR-mediated Fas translation. Our findings suggest that AFP is involved in the pathogenesis and chemosensitivity of HCC and that blockade of AFP may be a promising strategy to treat advanced HCC.

## Introduction

Hepatocellular carcinoma (HCC) is one of the most common malignances worldwide, causing ~750,000 deaths annually^1^. Multiple risk factors, including hepatitis virus infection, aflatoxin B1 exposure and alcohol consumption, have been identified as causes of HCC pathogenesis^2^. Although an increasing number of diagnostic and therapeutic strategies have been developed, the prognosis of HCC patients remains poor^3^. This is mainly due to the key genes and molecular mechanisms involved in HCC pathogenesis and development remaining largely unclear^3,4^.

Alpha fetoprotein (AFP) is a glycoprotein synthesized in the fetal yolk sac and liver during the gestational period. Since the discovery of this tumor-associated fetal protein in the mid-1960s, AFP has been widely used in clinical practice^5^. An abnormal increase in the plasma AFP level in adults is considered to be a hallmark of pathological conditions, including HCC, yolk sac tumors, and gastric carcinoma^6^. AFP is reactivated in more than 70% of HCC patients, and serum AFP has been considered the ‘gold-standard’ biomarker for clinical liver cancer diagnosis for the past several decades^5^. AFP is associated with fetal defects and malformations, and abnormal levels of AFP in maternal serum have been used as an indicator of spina bifida or Down’s syndrome in fetal screening^7,8^. In recent years, AFP has been used to monitor HCC treatment and to guide therapeutic decision-making and prognostic analysis^9^. In addition to its potential in clinical applications, the physiological, chemical, and immunological properties of AFP have also been extensively characterized. AFP acts as a carrier that binds and transports unsaturated fatty acids, estrogens, vitamin A, steroids, and flavonoids^6,7^. AFP has the capability to influence immunity by interacting with macrophages or inhibiting natural killer cells^10,11^. However, the biological functions of AFP have received comparatively little attention^5^. Several studies have found that AFP is associated with female infertility and that AFP deficiency in mice causes dysfunction of the hypothalamus-pituitary axis and anovulation^12,13^. Although efforts have been made to determine the role of AFP in cancer, the findings from different time periods are contradictory. Early studies in the 1980s demonstrated that AFP was capable of inhibiting cancer cell growth and differentiation^14,15^. AFP-derived peptide fragments have been developed to suppress the growth of human cancer cells^16^. These early studies suggest that AFP may be a potential tumor suppressor^16,17^. Studies over the next two decades found that AFP could serve as a dual regulator of cell proliferation and that the enhancing or inhibitory effect of AFP on growth regulation depended on the concentration of AFP and concentrations of cytokines, hormones, and growth factors in the culture system^5,18,19^. It is noteworthy that the studies in these two periods tended to explore the functions of extracellular AFP protein and many of these studies were performed with breast cancer cells. During the last decade, increasing efforts have been made to determine the biological function of AFP in HCC, as AFP shows a close association with the high mortality rate of this cancer^9,20^. In several studies, administration of the AFP protein resulted in increased HCC cell proliferation in vitro^21,22^, and ectopic expression of AFP promoted the growth of HCC cells in nude mice^23^, suggesting that AFP may play a pro-oncogenic role in HCC tumor growth. These studies also proposed several possible molecular mechanisms to explain the growth-promoting function of AFP in HCC. In vitro studies suggested that extracellular AFP could affect the expression of certain oncogenes and the activation of Fas receptor-Fas ligand (FasL) signaling pathway, shielding HCC cells from apoptosis or allowing escape from immune surveillance^21,22,24^. Based on an analysis of clinical specimens, Parpart et al. proposed that extracellular AFP inhibits the transcription of miR-29 and alters the epigenetic status of tumor cells in HCC patients^23^. In recent years, increasing evidence has indicated that cytoplasmic AFP plays role in the regulation of growth signaling pathways in HCC. Studies have shown that cytoplasmic AFP can promote the degradation of PTEN and activate the AKT/mTOR signaling pathway^25^. The interaction between AFP and retinoic acid receptor (RAR) disrupts the entry of RAR into the nucleus and alters the expression of some tumor-associated genes, such as Fn14 and GADD153^26,27^. Although all these pieces of evidence suggest that AFP may play important roles in HCC, the precise functions of AFP in hepatocarcinogenesis and associated underlying mechanism remain incompletely understood^28^. In addition, current findings are insufficient to explain the critical clinical significance of AFP in HCC prognostic analysis and therapy.

In this study, we generated mice lacking AFP to clarify its functional roles in hepatocarcinogenesis. Our data confirmed the pro-oncogenic role of AFP in HCC progression and uncovered a novel antiapoptotic mechanism connecting AFP to human antigen R (HuR)-mediated Fas mRNA translation. Our study suggests that AFP is involved in the pathogenesis and chemosensitivity of HCC and that blockade of AFP may be a promising strategy to treat advanced HCC displaying AFP overexpression.

## Materials and methods

### Reagents and antibodies

The following reagents were used in this study. Sorafenib, oxaliplatin, and paclitaxel were purchased from Selleck (Selleck, China). Cisplatin, irinotecan, and 5-fluorouracil (5-Fu) were purchased from Sigma (Sigma-Aldrich, USA). Targeted specific siAFPs were synthesized by Genepharma (Shanghai, China), and the sequences are listed in Supplementary Table 1. SiFas (sc-29311) was purchased from Santa Cruz Biotechnology. For Western blot analysis, the following antibodies were used. Anti-PARP (#9532), anti-caspase 3 (#9662), anti-caspase 9 (#9502), anti-myc-tag (#2278), anti-Cyto c (#11940), anti-β-actin (#4970 S) and anti-GAPDH (#2118) antibodies were purchased from Cell Signaling Technology. Anti-AFP (SC-166325), anti-Fas (SC-1204 and SC-8009), anti-FasL (SC-834), anti-FADD (SC-271748), anti-Bcl2 (SC-509), anti-Lamin A/C (sc-7292), and anti-Bax (SC-493) antibodies were purchased from Santa Cruz Biotechnology. An anti-caspase 8 antibody (#AF1650) was purchased from R&D Systems, anti- IR-β (ab69508) antibodies were purchased from Abcam, and an anti-HuR antibody (#07-468) was purchased from Millipore.

### Cell lines and cell culture

HepG2 and SNU475 cells were obtained from American Type Culture Collection (ATCC; USA). HuH7 cells were purchased from the Cell Bank of the Chinese Academy of Sciences (Shanghai, China). The HLE cell line was purchased from the CoBioer Inc. (Nanjing, China). All cells were cultured in the recommended medium (DMEM for HepG2 and HuH7 cells and RPMI-1640 medium for HLE and SNU475 cells) supplemented with 10% fetal bovine serum (FBS) and 1% penicillin and streptomycin (p/s) at 37 °C in 5% CO_2_. The cell lines were authenticated recently, and no mycoplasma contamination was detected.

### Plasmid construction

The pCMV6-XL5-AFP plasmid was purchased from Origene (Beijing, China). The AFP CDS fragment was cut from this plasmid and then subcloned into the pcDNA3.1 vector (Invitrogen). Full-length human Fas and HuR cDNA templates were amplified from HEK293 cells using a One-Step RT-PCR System kit (Invitrogen) and subcloned into the pcDNA3.1 vector.

### Transient and stable transfections

Transfections were performed using Lipofectamine 2000 or Lipofectamine RNAiMAX (for siRNA) transfection reagent (Invitrogen) according to the manufacturer’s instructions. To obtain stable AFP transfectants, transfected HLE cells were grown in medium containing 100 μg/ml G418, and resistant clones were confirmed using Western blotting and quantitative real-time PCR (qRT-PCR).

### Patient specimens and tissue microarrays

Five pairs of AFP-negative or AFP-positive human HCC tissue samples were randomly collected from patients at The First Affiliated Hospital of Wenzhou Medical University (Wenzhou, China) who provided informed consent, and the study protocols were approved by the Institutional Ethics Committee of Wenzhou Medical University. High-density tissue microarrays (TMA) of human HCC clinical samples were obtained from a cohort of 95 patients (Cat. No. Z1-LVC1021), constructed and performed by Superbiotek Inc. (Shanghai, China).

### HCC mouse model

All animal experiments were performed according to the animal protocols approved by the Wenzhou Medical University Institutional Animal Care and Use Committee. Afp-heterozygous hepatocarcinogenesis reporter (HCR) mice^29^ were generated on the C57 BL/6j-129 background originally and backcrossed to C3H and C57BL/6 mice for five generations by standard genetic crosses, and the resulting offspring were then bred with each other to generate cohorts of Afp-deficient (*afp-/-*) and wild-type (*wt*) mice that were used in subsequent studies. Mice were bred in the Wenzhou Medical University Laboratory Animal Center and maintained in a specific pathogen-free environment. Mice (5 per cage) were provided free access to food and water and kept in a colony room on a 12:12-hr light/dark cycle. To induce hepatocarcinogenesis, male AFP-deficient (*afp-/-*) mice and wild-type (*wt*) littermates were subjected to a single intraperitoneal injection of DEN (Sigma-Aldrich) at a dose of 20 μg/g of body weight at 14 days after birth. DEN-treated C3H mice were sacrificed at 8 months of age, while DEN-treated C57BL/6 mice were sacrificed at 12 months of age. Livers were excised, and tumor nodules were counted, measured, and photographed. Tissue samples were either snap frozen in liquid nitrogen or fixed in 10% formalin for subsequent histological and immunohistochemical analyses.

### MTT and clonogenic assays

Cell viability was analyzed by an MTT (Sigma) assay as described previously^30^. For colony formation assays, 1000 HLE cells stably overexpressing AFP or 5000 siAFP-transfected HuH7 or HepG2 cells were seeded in 6-well plates and maintained for 14 days in medium containing 5% FBS. To detect the drug cytotoxicities, HCC cells were plated in 6-well plates overnight in triplicate. After treatment with the indicated concentrations of drugs for 48 h, the cells were cultured in drug-free medium for 14–18 days. At the end of the experiments, colonies (≥50 cells per colony) were counted after staining with a 0.5% crystal violet solution in 20% methanol.

### RTCA assay

Cell proliferation was monitored by using the RTCA-MP system (Acea Biosciences). Cells were seeded in an E-plate 16 and locked in an RTCA-MP device at 37 °C with 5% CO_2_. Measured changes in electrical impedance are presented as the cell index, which reflects cellular proliferation. The cell index was read automatically every 15 min, and the recorded curve is shown as the cell index ± SD.

### Xenograft tumor assay

Five-week-old male athymic nude mice (nu/nu) were purchased from the Vital River Experimental Animal Center (Beijing, China) and maintained under pathogen-free conditions. Mice were randomly divided into two groups. HuH7 cells (5 × 10^6^) stably infected with the indicated shAFP or nontargeted (shNC) lentivirus (GeneChem Co. Shanghai, China) were suspended in 100 μl of PBS and subcutaneously injected into the dorsal flanks of mice. Tumor volume was measured every 4 days with a caliper and calculated using the standard formula: V = A × B^2^ × 0.5326 (A = long axis and B = short axis). At the end of the experiments, the mice were sacrificed, and the tumors were harvested and photographed. All animals were maintained and used in accordance with the guidelines of the Institutional Animal Care and Use Committee of Wenzhou Medical University.

### Luciferase assays

The human Fas promoter (from −1780 bp to +1 bp) region was amplified from HEK293 cell genomic DNA and subcloned into the pGL3 basic vector (Promega) upstream of the luciferase gene. The full-length human (2.6 kb) or mouse (0.45 kb) Fas 3′-UTR was amplified from HEK293 cell or C57BL/6 mouse genomic DNA, respectively, and subcloned into a pGL3-control vector (Invitrogen). Cells were cotransfected with 50–100 ng of the indicated reporter construct and 2.0 ng of pRenilla. Twenty-four hours after DNA transfection, the cells were harvested and analyzed using a dual-luciferase reporter assay kit (Promega). Firefly luminescence signals were normalized according to the corresponding Renilla signals, resulting in the calculation of relative luciferase activity. Mean luciferase activity levels were derived from three independent experiments.

### qRT-PCR

Total RNA was extracted from fresh tissue samples or cells using TRIzol Reagent (Invitrogen), and reverse transcription was performed using a MLV-reverse transcriptase kit (Invitrogen). qRT-PCR was carried out with SYBR Green (Tiangen, China) in triplicate assays on an ABI 7500 Real-Time Detection system (Applied Biosystems) according to the manufacturer’s protocol. GAPDH was used as an internal standard. Experiments were performed in triplicate, and data were calculated using the 2^−ΔΔCt^ method. Primer sequences are listed in Supplementary Table 1.

### Apoptosis analysis

Cell apoptosis was analyzed by flow cytometry. The Annexin V-APC/7-AAD Apoptosis Detection Kit (BD Biosciences) was used in accordance with the manufacturer’s instructions. Briefly, HCC cells receiving different treatments (1 × 10^5^) were harvested, washed twice with cold PBS, and resuspended in a 1× binding buffer. Next, the cells were incubated with 5 μl of Annexin-APC and 5 μl of 7-AAD for 15 min at room temperature in the dark. The cells were immediately analyzed using a FACSCalibur flow cytometer (Becton Dickinson). All experiments were performed in triplicate. Apoptosis in tumor tissues was analyzed by a TUNEL assay, and the apoptotic cells were determined using an in situ apoptosis detection kit (KEYGEN, China) according to the manufacturer’s instructions. The frequency of apoptotic cells in a section of tumor sample was obtained by calculating the mean number of TUNEL-positive brown nuclei among the hepatocytes in five randomly selected fields viewed under a microscope at ×100 magnification.

### Western blot analysis

Cell lysis and protein concentration determination were performed as described previously^30^. Cytoplasmic and cell membrane proteins were isolated using NE-PER^™^ Nuclear and Cytoplasmic Extraction Reagents (Thermo Scientific) and Cell Surface Protein Isolation Kit (Pierce) according to the manufacturer’s instructions. For a Fas CHX-chase assay, HLE cells were treated with 500 nM cycloheximide (Sigma-Aldrich), and total protein was harvested at the indicated time points. The proteins were separated using SDS-PAGE and transferred to polyvinylidene difluoride membranes (Bio-Rad). After blocking in 5% milk in TBST (0.1% Tween-20), the membranes were incubated with corresponding primary antibodies followed by horseradish peroxidase (HRP)-conjugated secondary antibodies. Protein bands were visualized with an Immun-Star HRP Chemiluminescence kit (Bio-Rad). Densitometry quantified by ImageJ software was used to analyze the target bands.

### Histology and immunohistochemistry (IHC)

Tumor tissue samples were fixed in 10% formalin and embedded in paraffin. Five-micron-thick sections were cut, deparaffinized in xylene, dehydrated in graded ethanol and then stained with hematoxylin and eosin (H&E). For IHC, 4 μm-thick consecutive sections were cut and mounted on glass slides. After deparaffinization, the tissue sections underwent antigen retrieval, were washed and were treated with a peroxidase blocking solution (Santa Cruz). Then, the sections were incubated with an anti-AFP (ab46799, Abcam) or anti-Fas antibody (SC-8009, Santa Cruz). After washing with PBS, the sections were incubated with a biotinylated secondary antibody and HRP-streptavidin complex (Santa Cruz). Color was developed using a diaminobenzidine substrate. All sections were counterstained with diluted hematoxylin. Immunohistochemical staining of tissue microarrays was performed with specific antibodies against AFP (ab46799, Abcam) and Fas (SC-8009, Santa Cruz). After immunohistochemical staining, the tissue microarray chips were digitally scanned, and the levels of AFP and Fas were scored semiquantitatively based on staining intensity and distribution using the immunoreactive score (IRS). Briefly, the final IRS was determined by multiplying the intensity score by the extent of positivity score of the stained cells. The intensity score was assigned as follows: 0 = negative; 1 = weak; 2 = moderate; and 3 = strong. The positivity score was defined as 0 = 0%; 1 = 0–25%; 2 = 25–50%; 3 = 50–75%; and 4 = 75–100%. The correlation between the AFP and Fas IRSs was determined by the Pearson correlation test.

### Co-IP

Co-IP was performed as described previously^30^. Briefly, cells were washed with ice-cold PBS and lysed in an immunoprecipitation assay buffer (50 mM Tris/HCl, pH 7.5, 150 mM NaCl, 1% Triton X-100, 1 mM EDTA and 10% glycerol) supplemented with the Protease/Phosphatase Inhibitor Cocktail (Cell Signaling Technology). The lysates were incubated on ice for 20 min and centrifuged at 12,000 rpm for 20 min at 4 °C. The cell lysates were incubated with an anti-AFP (SC-166325, Santa Cruz) or anti-HuR (#07–468, Millipore) antibody for 4 h, and an IgG antibody was used as a negative control. Following a washing step with PBS, protein G-sepharose beads (GE healthcare) were mixed with the whole-protein lysates and incubated for 1 h at 4 °C. The beads were washed four times with the immunoprecipitation assay buffer, suspended in Laemmli buffer, and boiled for 5 min. The eluted Co-IP lysates were analyzed by Western blotting.

### Immunofluorescence imaging

Immunofluorescence analysis was performed as described previously^31^. Briefly, 1 × 10^5^ HCC cells were seeded on coverslips and cultured for 24 h. After gentle washes with PBS, the cells were fixed with 4% formaldehyde for 20 min and permeabilized with 0.5% Triton X-100 in PBS for 10 min at room temperature. After blocking with 2% BSA in PBS-0.1% Triton X-100 for 10 min, the cells were incubated overnight with an anti-AFP mouse antibody (1:100 dilution; SC-166325, Santa Cruz) and an anti-HuR rabbit antibody (1:200 dilution; #07–468, Millipore) at 4 °C. The cells were washed three times in PBS and then incubated with fluorophore-labeled secondary antibodies for 2 h at room temperature. The coverslips were mounted on glass slides in the presence of 4′,6-diamidino-2-phenylindole (DAPI) for nuclear staining, and cell images were recorded using a ZEISS confocal microscope.

### Data mining

Serum levels of AFP in HCC patients were obtained from a TCGA dataset (TCGA_LIHC_phenotype_clinicalMatrix-2016-04-27). High AFP expression was defined as a serum AFP level ≥4 mg/ml. Kaplan–Meier analysis was used to determine patient overall survival rates.

### Statistical analysis

Data are presented as the mean ± SD. Statistical analysis was performed using the SPSS statistical software program (SPSS20, IBM, USA). Survival curves were analyzed by the Kaplan–Meier log-rank test, and correlations were analyzed by the Pearson correlation test. A two-tailed Student’s *t* test was used to perform comparisons between different groups. Values of *P* < 0.05 were considered statistically significant.

## Results

### AFP plays a pro-oncogenic role in liver cancer progression

**Fig. 1:**
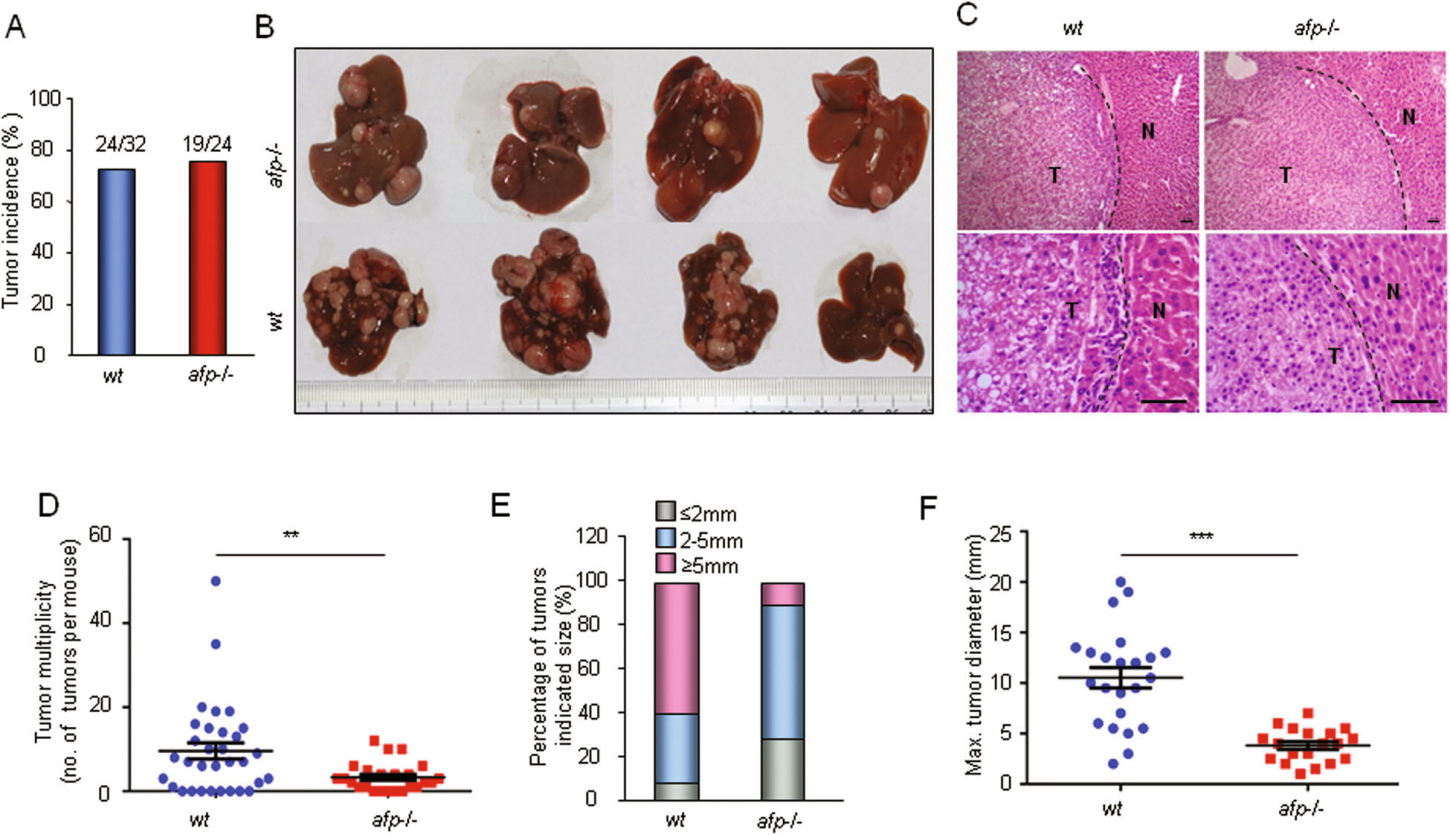
AFP accelerates DEN-induced liver tumor progression in C3H mice. **a** Liver tumor incidence in Afp-deficient (*afp-/-*; *n* = 24) and wild-type (*wt*, *n* = 32) C3H mice at 8 months after DEN injection. **b** Typical gross morphology of liver tumors from DEN-treated *afp-/-* or *wt* mice. **c** Representative microscopic features of HCC in hematoxylin and eosin (H&E)-stained liver sections from mice (Top, ×10 magnification; bottom, ×40 magnification; T: tumor; N: normal tissue). Scale bar, 100 μm. **d** Liver tumor numbers compared between *afp-/-* (*n* = 19) and *wt* (*n* = 24) mice. Data are expressed as the mean ± SD. **e** Sizes of liver tumors in *afp-/-* and *wt* mice. **f** Average maximal diameters of tumors compared between *afp-/-* and *wt* mice. *P < 0.05, **P < 0.01, and ***P < 0.001 versus *wt* mice.

Using a previously established HCR mouse model^29^, we generated an *Afp* gene-deficient mouse strain (*afp-/-*) (Supplementary Fig. 1) and investigated the role of *Afp* in hepatocarcinogenesis. Consistent with our previous report, homozygous *afp-/-* mice did not display any phenotypic or histological abnormalities when compared with their normal counterparts except that the females were sterile^29^. We initially examined the prevalence of diethylnitrosamine (DEN)-induced liver cancer in mice on the C3H genetic background. The results showed that there was no significant difference in the incidence rate of liver cancer between the wild-type (*wt*) and *afp-/-* mouse cohorts (Fig. 1a). However, compared with the wild-type mice, the *afp-/-* mice demonstrated significantly reduced tumor multiplicity (Fig. 1b-d) and much smaller tumor sizes (Fig. 1e-f). These results indicate that depleting Afp does not affect the initiation of liver cancer but suppresses tumor growth in individual C3H mice. Intriguingly, we observed no significant differences in liver cancer incidence, multiplicity, or tumor size between wild-type and *afp-/-* mice on the C57BL/6 genetic background (Supplementary Fig. 2). To ascertain the cause of this difference, we examined the protein expression levels of AFP in tumors from individual C3H and C57BL/6 mice. The results indicated that the majority of the liver tumors (~80%) from the C3H mice presented moderate to strong AFP protein expression, but only 15% of the tumors from the C57BL/6 mice showed AFP expression (Supplementary Fig. 3). Therefore, these data confirm the pro-oncogenic role of AFP in liver cancer progression.

**Fig. 2:**
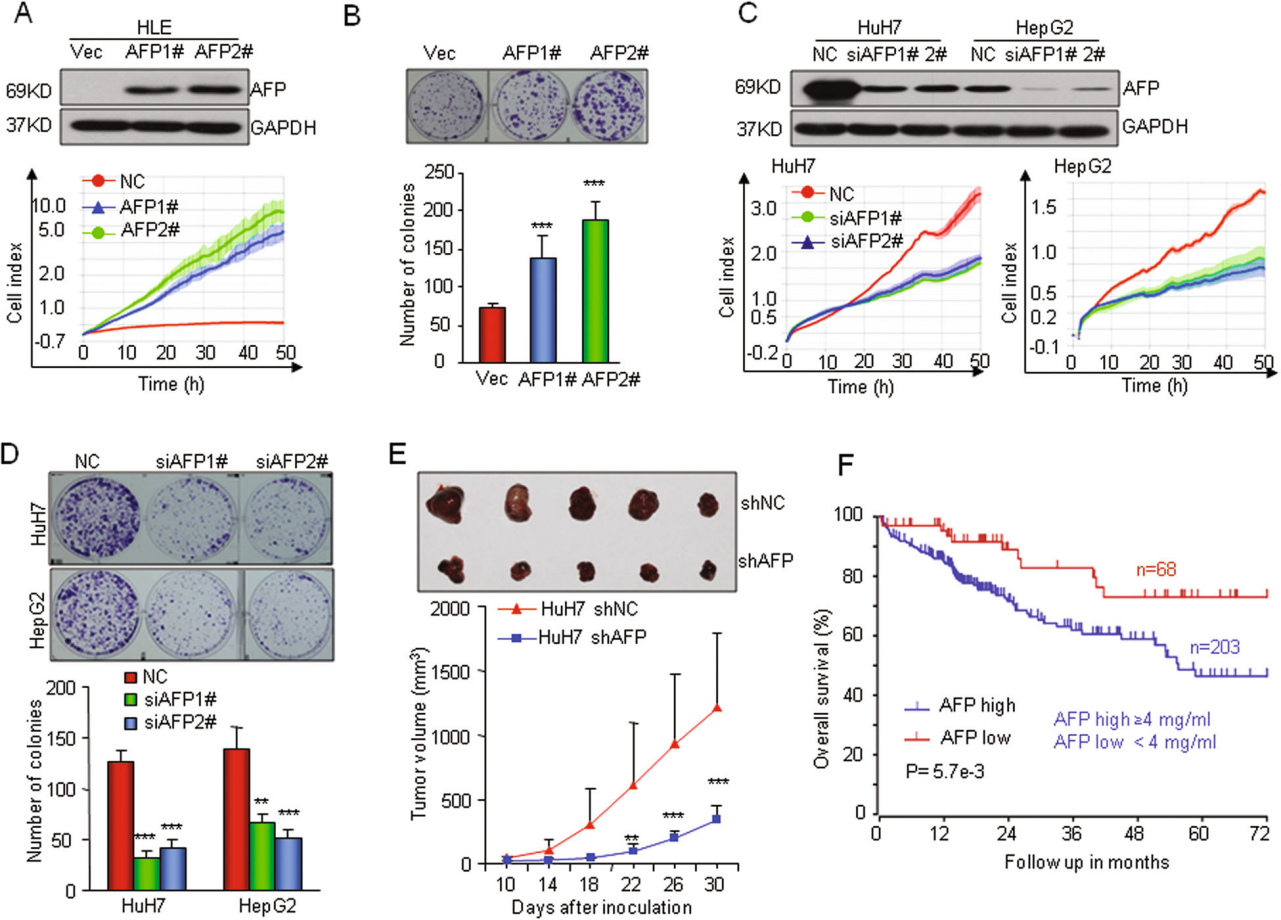
AFP promotes human HCC cell proliferation in vitro and tumorigenesis in vivo. **a** Ectopic overexpression of AFP promoted HLE cell growth. HLE cell lines with stable AFP expression (AFP1# and AFP2#) were established and verified by Western blotting (top). Cell proliferation was monitored by an RTCA assay. **b** Ectopic overexpression of AFP increased the clonogenic formation of HLE cells. **c–d** Knocking down AFP expression in HuH7 and HepG2 cells reduced the capabilities of cell proliferation and clonogenicity. The cells were transfected with a nontarget control (Non) or siRNAs against AFP (siAFP1# and siAFP 2#). **e** AFP knockdown suppressed tumor growth in nude mice. HuH7 cells were stably infected with a lentivirus containing an shRNA that interfered with AFP expression (shAFP) or a nontarget shRNA (shNC). The lower panel shows the tumor volume changes at 10 days after inoculation. The upper panel shows tumors that were dissected and photographed at 30 days post inoculation (*n* = 5). **f** Kaplan–Meier analysis compared the overall survival rates of 271 HCC patients from the TCGA database stratified by their serum AFP concentration. High AFP expression was defined as a serum AFP level ≥ 4 mg/ml. *P < 0.05, **P < 0.01, and ***P < 0.001 versus control.

To determine the pro-oncogenic role of AFP in human HCC, we first stably overexpressed the AFP gene in HLE cells, which do not express AFP. Real-time cellular analysis (RTCA) and a clonogenic assay demonstrated that overexpression of AFP significantly promoted HLE cell proliferation (Fig. 2a-b). We next knocked down AFP expression in HuH7 and HepG2 cells, which exhibit high levels of basal AFP expression. This in vitro experiment indicated that silencing AFP markedly inhibited cell growth (Fig. 2c-d and Supplementary Fig. 4A). Furthermore, knocking down AFP expression significantly suppressed the tumorigenicity of HuH7 cells in nude mice (Fig. 2e). Taken together, these results indicate that AFP drives human HCC cell growth and tumorigenicity. Consistent with the pro-oncogenic role of AFP in HCC cells, HCC patients with high serum levels of AFP had a significantly lower overall survival rate than those with low AFP levels, as determined by analyzing data from The Cancer Genome Atlas (TCGA) database (Fig. 2f).

### AFP promotes HCC growth by suppressing the Fas-mediated apoptotic pathway

**Fig. 3:**
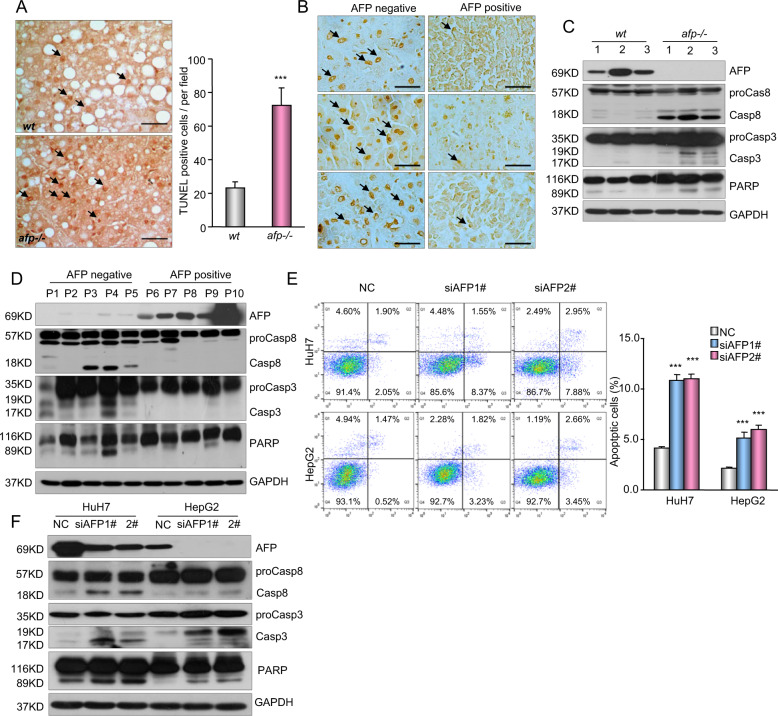
AFP inhibits cell apoptosis in HCC. **a** Cell apoptosis in AFP-positive wild-type (*wt*) and AFP-deficient (*afp-/-*) liver tumors from C3H mice was examined by TUNEL staining. Left panel, representative images of the TUNEL assay are shown. Right panel, the frequency of apoptotic cells was calculated and is shown as the mean ± SD of 5 mice per group. *Scale bars*, 50 μm. ***P < 0.001 versus *wt*. **b** Cell apoptosis in AFP-positive and AFP-negative human HCC specimens was examined by TUNEL staining. Data were analyzed and are shown as the mean ± SD of five patients per group. *Scale bar*, 50 μm. **c** A Western blot shows the expression of caspase-3, caspase-8 and PARP in AFP-positive *wt* and *afp-/-* mouse liver tumors. **d** A Western blot shows the expression of apoptosis-related proteins in AFP-negative and AFP-positive human HCC specimens. **e** The effects of AFP knockdown on HCC cell apoptosis were examined. HuH7 and HepG2 cells transfected with a nontarget control (NC) or siRNAs against AFP (siAFP1# and siAFP2#) were analyzed by flow cytometry. Apoptotic cells (%, Q2 + Q4) are reported as the mean ± SD of three replicate experiments. ***P < 0.001 versus the NC. **f** The effects of AFP knockdown on the activation of caspase-3, caspase-8 and PARP were evaluated. HuH7 and HepG2 cells were transfected with the NC or siRNAs against AFP (siAFP1# and siAFP2#).

Resistance to apoptosis is a key event in tumor development^32^. A TUNEL assay showed that apoptotic cells were significantly more common in *Afp*-deficient mouse liver tumors than in wild-type *Afp*-positive liver tumors (Fig. 3a), and the same phenomenon was observed in human HCC tissue samples (Fig. 3b). In agreement with these observations, the activation of apoptosis-related proteins, including caspase 3, caspase 8, and PARP, was markedly increased in AFP-negative tumor samples (Fig. 3c-d). These data suggest that AFP is inversely associated with cell apoptosis in HCC tumors. Knocking down AFP expression in HepG2 and HuH7 cells induced cell apoptosis and simultaneously enhanced caspase 3, caspase 8, and PARP activation (Fig. 3e-f). The opposite pattern was observed in HLE cells following AFP overexpression (Supplementary Fig. 4B-C). Moreover, AFP had little effect on the levels of intrinsic apoptosis markers, such as Bax, Bcl2, and Cyto c, in both HCC cells and tissue samples (Supplementary Fig. 4D-F). Together, these data suggest that AFP has a negative influence on cell apoptosis and that inhibiting the extrinsic apoptotic pathway may exert a principal effect on HCC cell growth and tumor progression.

**Fig. 4:**
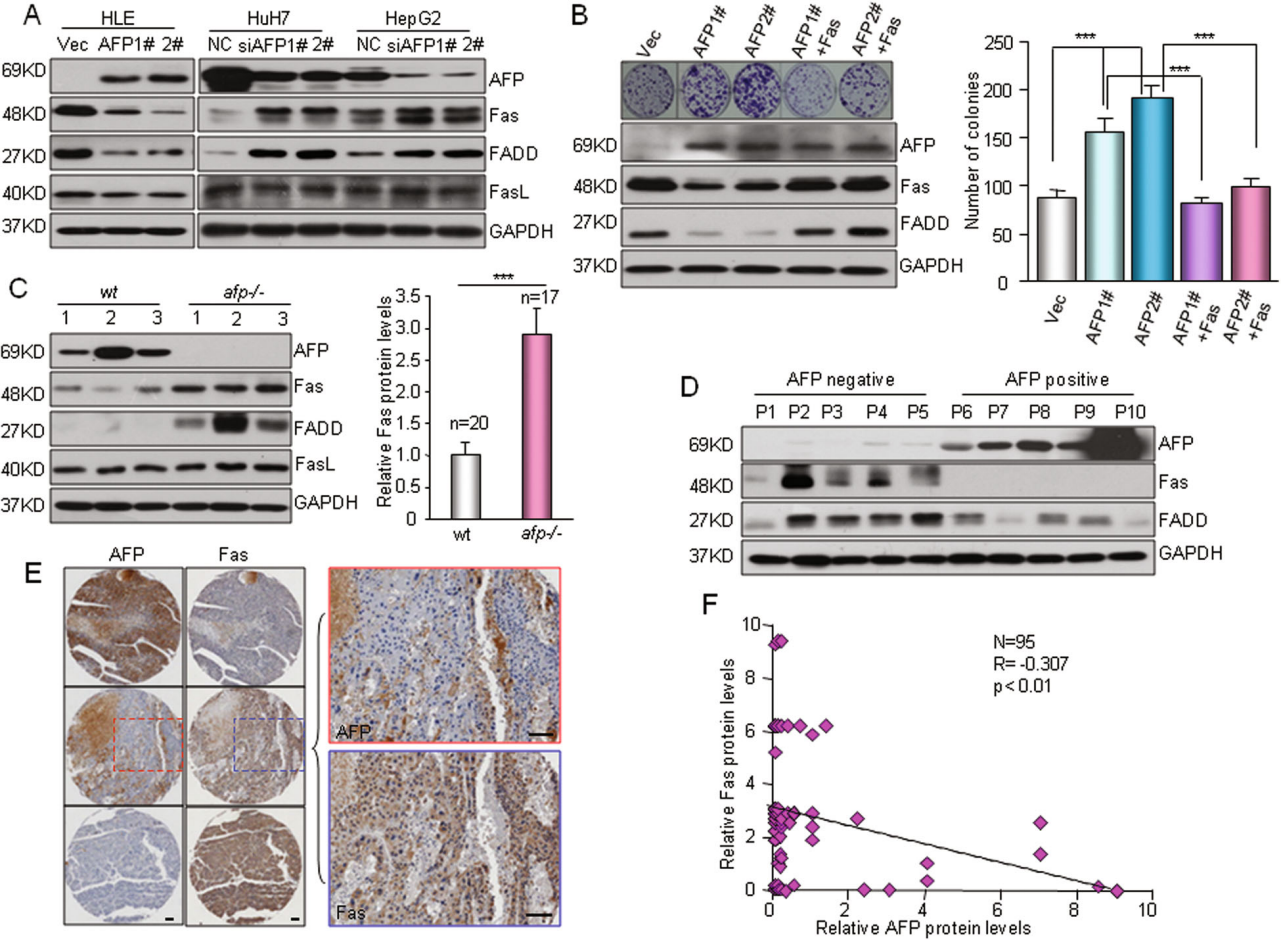
AFP suppresses HCC apoptosis via the Fas-FADD signaling pathway. **a** Western blotting showed the effects of AFP overexpression or knockdown on the expression of Fas/FasL/FADD. Left, HLE cells were stably transfected with AFP expression plasmids (AFP1# and AFP2#) or an empty vector (Vec). Right, HuH7 and HepG2 cells were transfected with a nontarget control (NC) or siRNAs against AFP (siAFP1# and siAFP2#). **b** Restoration of Fas expression counteracted AFP-enhanced cell growth. Fas was re-expressed in the HLE cells stably expressing AFP (AFP1# and AFP2#). Cell growth was determined by a clonogenicity assay. The expression levels of AFP and Fas/FADD were detected by Western blotting (left). ***P < 0.001. **c** The expression of AFP and Fas/FADD in AFP-positive wild-type (*wt*) and AFP-deficient (*afp-/-*) mouse liver tumors was determined. Left panel, representative bands of a Western blot are shown. Right panel, semiquantitative analysis of the Fas protein levels in AFP-positive *wt* (*n* = 20) and *afp-/-* (*n* = 17) mouse liver tumors is shown. Detailed Western blot data can be found in the Supplementary figures. ***P < 0.001 versus *wt*. **d** A Western blot shows the expression of AFP and Fas/FADD in human HCC specimens. **e** The protein expression of AFP and Fas in HCC patients was examined. A tissue microarray containing 95 primary human HCC specimens was used to evaluate the AFP and Fas protein levels by IHC, and representative images from three specimens are shown. *Scale bar*, 100 μm. **f** The correlation between the AFP and Fas proteins in HCC specimens was examined. Staining for AFP and Fas in the above tissue microarray was quantified, and the correlation between the AFP and Fas proteins was analyzed by the Pearson correlation test.

Fas/FADD activation is a key step in initiating the extrinsic apoptotic program^33^. Knocking down AFP expression resulted in significant increases in Fas/FADD levels in HuH7 and HepG2 cells, whereas overexpressing AFP in HLE cells suppressed Fas/FADD expression (Fig. 4a). Restoration of Fas expression completely nullified the growth-promoting effect of AFP on HLE cells (Fig. 4b). In addition, ectopic expression of Fas significantly inhibited HCC cell proliferation (Supplementary Fig. 5A-B). Together, these results indicate that AFP exerts its growth-promoting effect on HCC cells by suppressing the Fas-mediated extrinsic apoptotic pathway. In mouse liver tumor samples, we observed that high levels of AFP were usually accompanied by downregulated Fas expression (Fig. 4c and Supplementary Fig. 6A-B). Surprisingly, AFP had no effect on Fas ligand (FasL) expression in HCC cells, and the AFP level did not correlate with the FasL level in liver tumors (Fig. 4a and Supplementary Fig. 6B). Markedly reduced Fas expression was also observed in AFP-positive HCC cells and human tumor samples (Fig. 4d and Supplementary Fig. 6C-D). Immunohistochemical staining of a tissue microarray containing a cohort of 96 primary human HCC specimens showed that most of the HCC sections with high AFP expression exhibited low Fas expression, whereas those with low AFP expression showed strong Fas expression (Fig. 4e and Supplementary Fig. 6E). Spearman rank correlation analysis indicated a negative relationship between AFP and Fas protein expression in HCC patients (Fig. 4f, *R* = −0.307, *P* < 0.01). Collectively, these data imply that AFP-mediated Fas downregulation and apoptosis inhibition are significant in HCC patients.

### AFP inhibits Fas expression through HuR-mediated translational regulation

**Fig. 5:**
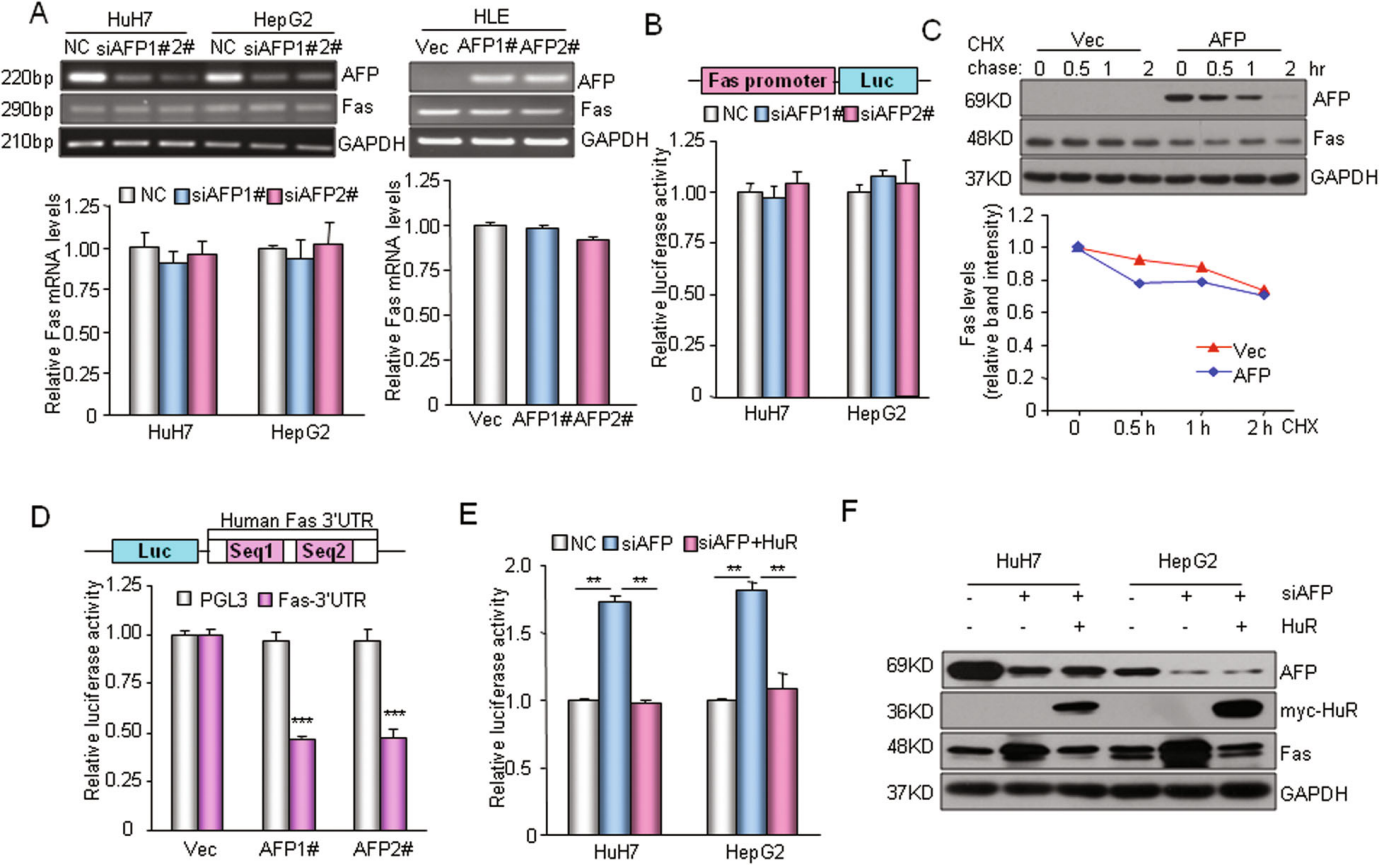
AFP translationally regulates Fas expression. **a** qRT-PCR analysis of Fas mRNA levels following AFP knockdown in HuH7 or HepG2 cells (left panel) or AFP overexpression in HLE cells (right panel). **b** Effect of AFP knockdown on the activity of a luciferase reporter bearing the human Fas promoter region. **c** Cycloheximide (CHX)-chase assay. The AFP protein level was measured by Western blotting. HLE cells without (Vec) or with AFP expression were treated with CHX (500 nM) for the times indicated. Data were normalized to the GAPDH and zero-time control data. **d** AFP-mediated suppression of Fas 3′-UTR reporter activity. A schematic representation of the luciferase reporter constructs with the human Fas 3′-UTR and identified HuR-binding sequences Seq1 and Seq2 is shown (top). A luciferase reporter construct was transfected into HLE cells stably expressing AFP (AFP1# and AFP 2#) or a control vector (Vec). ***P < 0.001 versus the control (Vec). **e** Elimination of siAFP-mediated human Fas 3′-UTR reporter activation by HuR. **f** Elimination of siAFP-mediated Fas/FADD protein enhancement in HuH7 and HepG2 cells by HuR. **P < 0.01.

Next, we explored the molecular mechanism involving AFP in the regulation of Fas protein expression. Silencing AFP in HepG2 or HuH7 cells had little effect on the Fas mRNA level, and overexpressing AFP also failed to downregulate Fas mRNA transcription in HLE cells (Fig. 5a). A dual-luciferase reporter assay showed that AFP did not affect Fas promoter activity (Fig. 5b). These data indicate that the regulation of Fas by AFP does not occur at the transcriptional level. Moreover, a cycloheximide (CHX)-chase assay showed that AFP did not affect the degradation of the Fas protein (Fig. 5c). Interestingly, AFP remarkably suppressed the expression of a luciferase reporter gene containing the 3′-untranslated region (3′-UTR) of the human or mouse Fas mRNA transcript (Fig. 5d-e and Supplementary Fig. 7A). These data suggest that AFP may suppress the expression of Fas via its 3′-UTR. In HepG2 and HuH7 cells, enhanced Fas protein expression or Fas 3′-UTR reporter activity caused by interference with AFP was completely eliminated by overexpressing the RNA-binding protein HuR in these cells (Fig. 5e-f). Overexpressing HuR in the three HCC cell lines also remarkably suppressed the protein level of Fas and the activity of the luciferase reporter containing the Fas 3′-UTR (Supplementary Fig. 7B-D) but had no effect on total Fas mRNA levels (Supplementary Fig. 7E). HuR has been shown to bind to the 3′-UTR of Fas mRNA and repress Fas translation in HCC cells^34^. Taken together, these results suggest that AFP suppresses Fas expression through HuR-mediated inhibition of translation.

### AFP-HuR interaction increases the distribution of HuR in cytoplasm

**Fig. 6:**
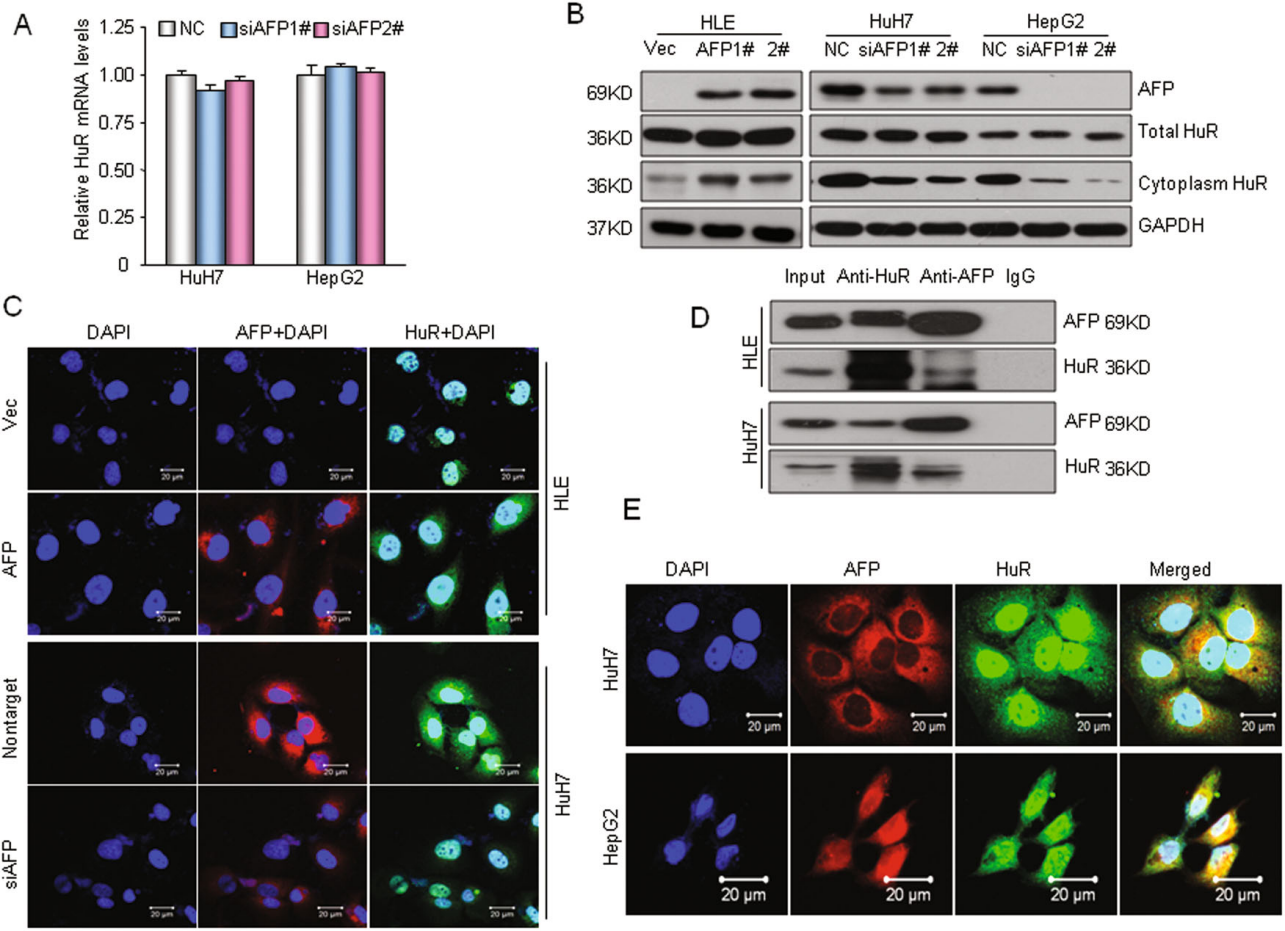
AFP-HuR interaction increases the HuR distribution in the cytoplasm. **a** qRT-PCR was used to analyze HuR mRNA levels in HuH7 and HepG2 cells following siRNA-mediated AFP knockdown. **b** Western blotting showed the effects of AFP overexpression or knockdown on total and cytoplasmic HuR protein levels. Left panel, HLE cells were stably transfected with an AFP expression plasmid. Right panel, AFP expression was knocked down in HuH7 and HepG2 cells by siRNA-mediated interference. **c** The AFP-altered cellular HuR distribution was detected by immunofluorescence staining and confocal microscopy imaging. Up, AFP was overexpressed in AFP-negative HLE cells; bottom, AFP was silenced in HuH7 cells. Scale bar, 20 μm. **d** Coimmunoprecipitation was used to analyze the HuR and AFP interaction in HCC cells. HLE cells were stably transfected with the AFP expression plasmid. **e** The colocalization of AFP and HuR in the HuH7 or HepG2 cell cytoplasm was captured by confocal microscopy. Scale bar, 20 μm.

To explore the mechanism underlying the regulation of HuR by AFP, we first examined the mRNA and protein expression of HuR following AFP manipulation. Silencing AFP did not change the HuR mRNA level in HuH7 or HepG2 cells (Fig. 6a). AFP also had no remarkable effect on total HuR protein levels in HCC cells (Fig. 6b). Interestingly, overexpressing AFP in HLE cells increased the cytoplasmic HuR protein level, whereas knocking down AFP expression in HuH7 or HepG2 cells markedly decreased cytoplasmic HuR protein levels (Fig. 6b). These results suggest that AFP alters the distribution of the HuR protein in the cytoplasm. HuR is an RNA-binding protein that is predominantly located in the cell nucleus, and transient translocation between the nucleus and cytoplasm does occur^35^. Indeed, AFP-mediated HuR cytoplasmic accumulation was confirmed by immunofluorescence staining (Fig. 6c). To further investigate the mechanism by which AFP modifies HuR cellular localization, we performed coimmunoprecipitation (CoIP) analysis. Ectopically expressed AFP pulled down endogenous HuR in HLE cells (AFP negative), and endogenous AFP and HuR in HuH7 cells coimmunoprecipitated reciprocally (Fig. 6d). Moreover, partial colocalization of AFP and HuR was observed in the cytoplasm of HuH7 and HepG2 cells (Fig. 6e). Taken together, these results suggest that cytoplasmic AFP is able to physically bind to the HuR protein and that the AFP-HuR interaction increases the HuR distribution in the cytoplasm, thus suppressing Fas mRNA translation in HCC cells.

### Inhibition of AFP enhances the chemosensitivity of HCC cells

**Fig. 7:**
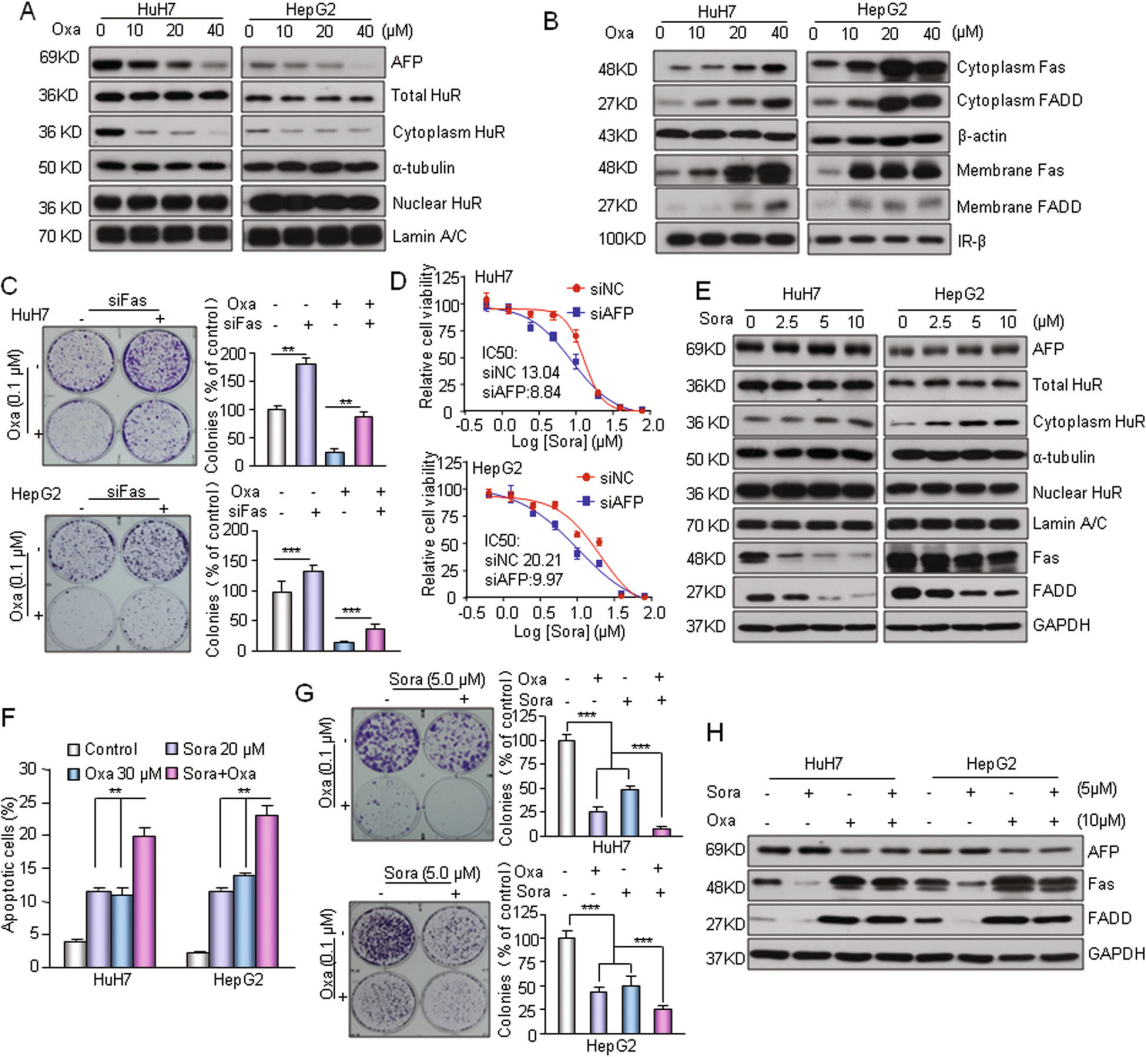
AFP-mediated cell apoptosis affects the chemosensitivity of HCC cells. **a** Oxaliplatin (Oxa) suppressed AFP expression and decreased the distribution of HuR in cytoplasm. Cells were treated with the indicated concentrations of oxaliplatin for 24 h. **b** Oxaliplatin activated the Fas/FADD apoptotic pathway in AFP-positive HCC cells. **c** Knocking down Fas expression attenuated oxaliplatin-induced cytotoxicity. Cell growth was determined by a clonogenic assay. Cells were transfected with siFas or a nontarget siRNA and reseeded in a 6-well plate for 12 h. Then, the cells were incubated with oxaliplatin (0.1 μM) for 40 h and allowed to grow into colonies for 14 days. **d** Knockdown of the AFP gene in HuH7/HepG2 cells resulted change in sensitivity to sorafenib. The AFP gene was knocked down by siRNA and the viability of cells was assessed by MTT assay. **e** Sorafenib enhanced cytoplasmic HuR levels and suppressed the activation of Fas/FADD in HuH7 and HepG2 cells. Cells were treated with the indicated concentrations of sorafenib for 24 h. **f** Sorafenib combined with oxaliplatin had a synergistic effect on the induction of apoptosis. Apoptotic cells (%, Q2 + Q4) are reported as the mean ± SD of three replicate experiments. **g** Combination treatment with sorafenib and oxaliplatin synergistically inhibited HuH7 and HepG2 cell growth. Cells were plated overnight, exposed to sorafenib and oxaliplatin individually or in combination at the indicated concentrations for 40 h and allowed to grow into colonies for 14 days. **h** Oxaliplatin in the combination treatment reversed the inhibitory effect of sorafenib on Fas/FADD activation. **P < 0.01 and ***P < 0.001.

AFP is generally considered to be a useful indicator for prognostic analysis and therapeutic evaluation^9^. Whether AFP inhibition plays a direct role in the treatment of HCC remains ambiguous. By testing several clinical anticancer drugs including oxaliplatin, 5-Fu and paclitaxel, we found that oxaliplatin dose dependently suppressed the mRNA and protein levels of AFP in AFP-positive HepG2 and HuH7 cells and simultaneously decreased the accumulation of HuR in cytoplasm (Fig. 7a and Supplementary Fig. 8A-B). Oxalipaltin also strongly activated the Fas/FADD apoptotic cascade by increasing the expression of Fas in cytoplasm and cell membrane (Fig. 7b and Supplementary Fig. 8C-D). Interestingly, oxaliplatin-induced Fas upregulation was not observed in two AFP-negative HCC cell lines (Supplementary Fig. 8E). Moreover, oxaliplatin did not induce further Fas/FADD activation when AFP was presilenced in HepG2 cells (Supplementary Fig. 8F). These results suggest that oxaliplatin-induced AFP downregulation functions in activating the HuR-mediated Fas/FADD apoptotic pathway in AFP-positive HCC cells. Silencing Fas markedly attenuated oxaliplatin-induced cell growth arrest (Fig. 7c). AFP knockdown resulted in increased sensitivity to sorafenib in HuH7 and HepG2 cells (Fig. 7d). In addition, oxaliplatin showed significantly stronger cytotoxicity and apoptosis induction capabilities in AFP-positive HuH7 and HepG2 cells than in AFP-negative HCC cells (Supplementary Fig. 9A-B). Together, these data suggest that AFP-mediated Fas/FADD apoptotic signaling affects the chemosensitivity of HCC cells. Surprisingly, we found that sorafenib had no effect on the expression of cytoplasmic AFP but markedly suppressed the activation of Fas/FADD in HuH7 and HepG2 cells (Fig. 7e, Supplementary Fig. 9C). HuH7 and HepG2 cells demonstrated enhanced cytoplasmic HuR levels after sorafenib treatment, whereas HuR protein expression in whole cell and nuclear lysates did not significantly change between cells treated with sorafenib as compared with the untreated controls (Fig. 7e), indicating that sorafenib affect HuR translocation from the nucleus to the cytoplasm through mechanisms other than AFP. The combination of these two agents had a more potent effect than either drug alone in regard to the induction of apoptosis (Fig. 7f). More importantly, the combination treatment was obviously superior to the monotherapies in suppressing HuH7 and HepG2 cell growth (Fig. 7g). In addition, oxaliplatin completely reversed the inhibitory effect of sorafenib on Fas/FADD in the combination treatment (Fig. 7h). Taken together, these results suggest that the sorafenib-oxaliplatin combination has a synergistic antitumor effect on AFP-expressing HCC cells and that the AFP-mediated HuR/Fas/FADD apoptotic cascade performs a direct role in this process.

## Discussion

**Fig. 8:**
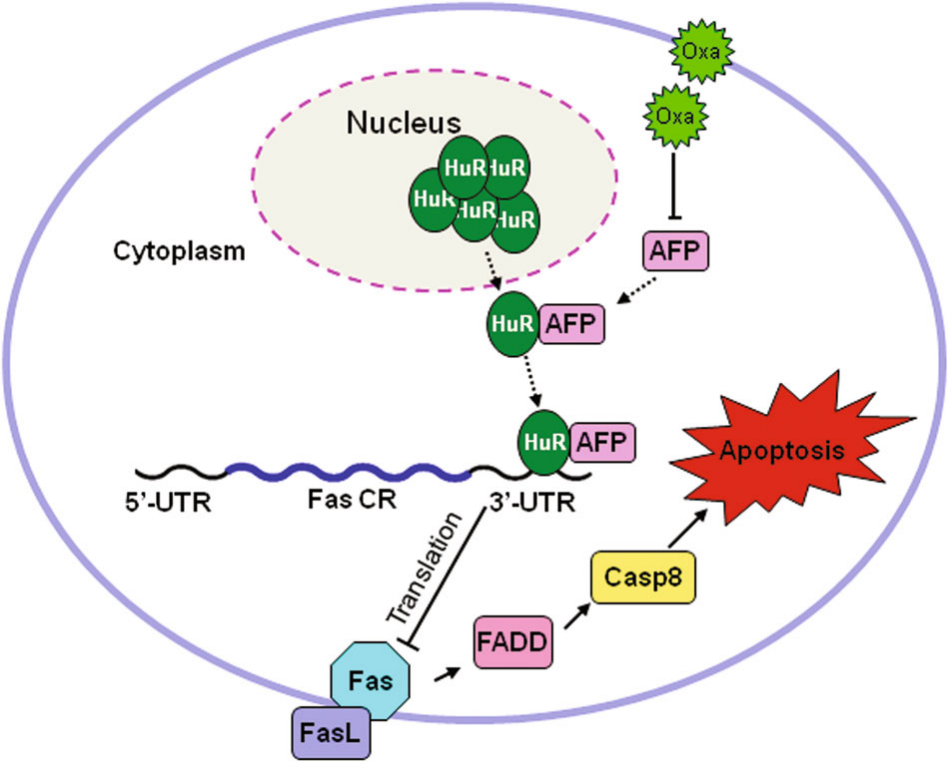
A model depicting how AFP regulating HCC progression. The interaction between AFP and HuR increases the accumulation of HuR in the cytoplasm and inhibits the translation of Fas mRNA, which leads to cancer cells escaping Fas/FADD mediated apoptosis and affects the chemosensitivity of HCC.

AFP has long been considered a biomarker for clinical liver cancer diagnostic and prognostic analyses^5^. Whether AFP plays direct roles in the development of HCC remains controversial. In this study, we confirmed the pro-oncogenic role of AFP in liver cancer development using an AFP-deficient mouse model. Furthermore, we revealed that the reactivation of AFP during hepatocarcinogenesis causes cancer cells to escape Fas/FADD-mediated apoptosis. The physical interaction between cytoplasmic AFP and HuR proteins increased the accumulation of HuR in the cytoplasm and subsequently suppressed Fas mRNA translation via an interaction with the 3′-UTR of Fas. Based on our experimental data, we propose a model in which cytoplasmic AFP regulates HCC progression and chemosensitivity (Fig. 8).

In recent years, increasing evidence has shown that AFP can function as a regulatory factor in HCC growth, but the findings have been contradictory^19,23,25,36^. A possible explanation is that it is difficult to determine whether extracellular or cytoplasmic AFP exerts the major effect on HCC development in vivo. In this study, we established an AFP gene-deficient mouse strain to investigate the function of AFP in hepatocarcinogenesis. Afp-/- mice are an ideal animal model that can mimic the human tumor microenvironment and faithfully reproduce the key biological behaviors of liver cancer^29^. In C3H-background mice, we demonstrated that depleting AFP did not affect liver cancer initiation but suppressed tumor progression. These findings suggest that AFP is not a cancer driver gene but is instead a growth-promoting factor in HCC progression. This conclusion is completely consistent with the characteristic that AFP is reactivated only after HCC occurs. Intriguingly, we found that AFP had no significant effect on tumor development in *afp-/-* mice on the C57BL/6 genetic background. The susceptibility of C57BL/6 and C3H mouse strains to DEN-induced hepatocarcinogenesis is quite different, but the cause of this difference is unclear. Previous studies have suggested that the high susceptibility of C3H mice is largely due to their high cell proliferation rate^37^. Through analysis of individual liver tumors, we found that the expression rate of AFP in liver tumors from C3H mice was much higher than that in those from C57BL/6 mice. These data, in turn, confirmed the growth-promoting effect of AFP on liver cancer and may also explain why C3H mice are more prone to DEN-induced hepatocarcinogenesis than C57BL/6 mice.

In the present study, we identified that suppression of the Fas/FADD apoptotic cascade is the key mechanism in AFP-promoted HCC progression. Apoptosis plays important roles in cell death, and escape from apoptosis is a key factor in the survival of malignant cells^32,38^. We first observed that overexpression of AFP in mouse and human HCC specimens was associated with a reduced rate of apoptosis and further confirmed the inhibitory effect of AFP on apoptosis in HCC cells. Our mechanistic study revealed that AFP significantly inhibited the Fas/FADD-mediated extrinsic apoptotic pathway. Fas is a well-known cell death-promoting factor, and the Fas/FasL system plays critical roles in liver cancer biology and chemotherapy^33,39^. It has been reported that knocking out Fas in mice reduces DEN-induced HCC formation by inhibiting the JNK pathway, suggesting Fas has oncogenic potential in mouse hepatocarcinogenesis^40^. However, the function of Fas in human HCC growth appears to be more complex. Our study demonstrated that overexpressing Fas suppressed HCC cell proliferation. These data suggest that Fas can also exert a growth-suppressive effect on human HCC. Previous studies found that extracellular AFP is able to promote the expression of FasL and inhibit the elevated Fas in Bel 7402 cells co-cultured with Jurkat cells, thus triggers the escape of liver cancer cells from immune surveillance^22,24^. We found that AFP had little effect on the FasL level but directly decreased the levels of Fas and its effector FADD in HCC cells. Furthermore, we demonstrated that coexpression of Fas with AFP completely eliminated AFP-promoted HCC cell growth. These results clearly indicate that Fas plays a tumor-suppressive role in AFP-promoted HCC growth. In normal human hepatocytes, Fas is constitutively expressed, and Fas-mediated apoptosis occurs continuously^41^. In this study, we demonstrated that depleting AFP increased the protein expression of Fas and activated the downstream extrinsic apoptotic pathway in both HCC cells and liver tumors. Fas expression has been found to be significantly reduced in aggressive forms of HCC and positively correlated with the apoptosis rate^39,42^. We found that the level of AFP was negatively correlated with that of the Fas protein and patient survival in a human HCC cohort. Our study, combined with those performed by others, suggests that the reactivation of AFP protects transformed hepatocytes from Fas/FADD-triggered apoptosis, thereby contributing to hepatocarcinogenesis and poor patient outcomes.

AFP is not a transcriptional regulator, and its regulation of gene expression usually requires a partner protein. Previous studies have reported that cytoplasmic AFP can interact with the PTEN and RAR proteins, affecting their stability or transcriptional activity^25,26^. In this study, we found that AFP affected neither Fas mRNA transcription nor Fas protein degradation. Intriguingly, we identified HuR as a novel AFP interaction partner for modulating Fas mRNA translation. HuR is an important posttranscriptional regulator that belongs to the ELAV family of RNA-binding proteins^43^. HuR regulates the expression of multiple genes involved in diverse cellular processes, ranging from cell development to cell survival and apoptotic death^44^. HuR can modify the stability or translation efficiency of mRNAs by binding to the AU-rich elements (AREs) in the 3′-UTR of its target mRNAs^45^. HuR functions as a repressor of Fas mRNA translation in HCC cells^34^. Bioinformatic analysis identified two ARE-binding motifs in both mouse and human Fas mRNA 3′-UTRs. Using a luciferase reporter assay, we demonstrated that HuR attenuated siAFP-induced mouse and human Fas 3′-UTR reporter activation. This finding suggests that AFP can regulate human and mouse Fas expression through the same posttranscriptional regulation mechanism. Intriguingly, we found that AFP did not change the total HuR protein level; it only altered the distribution of HuR in the cytoplasm. HuR is an RNA-binding protein mainly located in the nucleus. Redistribution of HuR from the nucleus to the cytoplasm is associated with functional alterations, especially in posttranscriptional gene expression regulation^35,45^. Our study found that AFP increased the presence of the HuR protein in the cytoplasm, which could enhance the binding of HuR to the 3′-UTR of Fas mRNA and eventually lead to translational suppression. In addition, we found that the AFP and HuR proteins were mutually immunoprecipitated and colocalized in the cytoplasm. The direct physical interaction between the AFP and HuR proteins explained the mechanism underlying AFP-mediated HuR accumulation in the cytoplasm. Collectively, our study unveiled a novel antiapoptotic mechanism connecting AFP to the HuR-mediated Fas/FADD apoptotic pathway, that is, the physical interaction between cytoplasmic AFP and HuR proteins increases the distribution of HuR in the cytoplasm, leading to the repression of Fas mRNA translation and subsequent inhibition of Fas/FADD-mediated cell apoptosis.

Serum AFP responses have been identified as significant prognostic factors in HCC patients^9^. In several exploratory clinical studies, researchers found that a high AFP level was a poor prognostic factor and that the reduction in serum AFP levels after sorafenib or oxaliplatin chemotherapy often predicted an improved therapeutic response and prolonged survival^46,47^. However, it is still not clear whether the decrease in the serum AFP level is the cause or result of the antineoplastic effects of these drugs. Our study indicated that sorafenib had no significant effect on the expression of AFP in HCC cells, suggesting that the decrease in the serum AFP level in HCC patients was most likely due to the regression of HCC tumors after sorafenib intervention. Intriguingly, our data showed that oxaliplatin significantly reduced the expression of AFP at the transcriptional level and that the downregulation of AFP expression directly contributed to the chemosensitivity to oxaliplatin and antitumor efficacy of oxaliplatin. Our findings suggest that AFP functions not only as a biomarker but also as a synergistic target for HCC treatment. In support of this hypothesis, we found that sorafenib combined with oxaliplatin produced a synergistic antitumor effect and that oxaliplatin-induced AFP silencing and Fas/FADD apoptotic signaling activation performed critical roles in this synergism. Consistent with our findings in HCC cells, the synergistic effects produced by combined sorafenib and oxaliplatin treatment have also been observed in several recent HCC clinical trials^48,49^. Taken together, these findings suggest that AFP is a potential HCC therapeutic target and that screening novel reagents or identifying potential measures to disrupt cytoplasmic AFP will produce direct benefits for the treatment of advanced HCC displaying AFP overexpression.

In conclusion, we confirmed the pro-oncogenic role of AFP in HCC progression. Cytoplasmic AFP regulates HCC growth and chemosensitivity through the Fas/FADD-mediated extrinsic apoptotic pathway. The interaction between AFP and HuR alters the cellular localization of HuR and then inhibits the translation of Fas mRNA. Our findings suggest that AFP can serve as a potential target in the development of therapeutics for liver cancer.

Supplementary information is available at Cell Death and Disease’s website

## Supplementary information

Supplementary Figure Legends

Supplementary figure 1

Supplementary figure 2

Supplementary figure 3

Supplementary figure 4

Supplementary figure 5

Supplementary figure 6

Supplementary figure 7

Supplementary figure 8

Supplementary figure 9

Supplementary Table 1
