# Supplementary material for: AFP promotes HCC progression by suppressing the HuR-mediated Fas/FADD apoptotic pathway
Source: Cell Death Dis. 2020 Oct 2;11(10):822. doi: 10.1038/s41419-020-03030-7 (PMC7532541; doi:10.1038/s41419-020-03030-7)
Supplement: Supplementary file 1 — Supplementary Figure Legends [file 41419_2020_3030_MOESM1_ESM.pdf]

Supplementary information: "AFP promotes HCC progression by suppressing the HuR-mediated Fas/FADD apoptotic pathway"

Chen et al.

Supplementary figure legends

Supplementary Figure 1 Generation of AFP-deficient mice. (A) Targeting strategy used to generate HCR and AFP-deficient (*afp-/-*) mice. The targeting cassette was inserted behind the alanine of the ATG translation start codon of the Afp gene to generate the primary HCR (Afp-heterozygous) mice. The HCR mice were bred with each other to generate cohorts of *afp-/-* mice. (B) PCR analysis of the genotypes of wild-type (*wt*), Afp-heterozygous (*afp+/-*) and *afp-/-* mice. The primers P1 (which binds in a region between the promoter and exon 1) and P2 (which binds in a region between exon 1 and exon 2) were designed to generate a 1000-bp PCR fragment for the wild-type allele, and the primer P3 (which binds in a region in the IRES) was designed to pair with P1 to generate a 720-bp fragment, which represents the interruption of the AFP gene (Afp deficiency, *afp-/-*). (C) Western blot analysis of AFP protein levels in livers isolated from newborn *wt*, *afp+/-* and *afp-/-* mice.

Supplementary Figure 2 Effect of AFP deficiency on DEN-induced hepatocarcinogenesis in C57BL/6 mice. (A) Incidence of HCC in Afp-deficient (*afp-/-*; n=21) or wild-type (*wt*; n=23) C57/B6 male mice at 12 months after DEN injection. (B) Typical gross morphology of liver tumors from DEN-treated *afp-/-* or *wt* mice. (C) Liver tumor numbers compared between *afp-/-* (*n* = 18) and *wt* (n=20) mice. Data are expressed as the mean ± SD. (D) Sizes of liver tumors in *afp-/-* and *wt* mice. (E) Average maximal diameters of tumors compared between *afp-/-* and wt mice. *P< 0.05, **P< 0.01, ***P< 0.001 versus *wt*.

Supplementary Figure 3 Difference in AFP expression patterns in DEN-induced

liver tumors between C3H and C57BL/6 mice. (A) Western blot analysis of AFP expression in liver tumors from wild-type C3H mice. Proteins isolated from the three largest tumors in each mouse were mixed together and analyzed by Western blotting. (B) Western blot analysis of AFP expression in liver tumors from wild-type C57BL/6 mice. (C) The percentage of AFP-positive liver tumors was compared between wild-type C3H and C57BL/6 mice.

Supplementary Figure 4 Effects of AFP on cell apoptosis and the apoptotic signaling pathway. (A) qRT-PCR analysis of the interference efficiency of siAFP. HuH7 and HepG2 cells were transfected with a nontarget control (NC) or siRNAs against AFP (siAFP1# and siAFP2#). (B) Ectopic overexpression of AFP inhibited HLE cell apoptosis. HLE cells stably transfected with AFP expression plasmids (AFP1# and AFP2#) or an empty vector (Vec) were analyzed by flow cytometry. Apoptotic cells (%, Q2+Q4) are reported as the mean ± SD of three replicate experiments. **$P< 0.01$ versus Vec. (C) The effects of AFP overexpression on the activation of caspase-3, caspase-8 and PARP in HLE cells were examined. (D) Western blotting showed the effects of AFP overexpression or knockdown on the expression of Bax, Bcl-2, and Cyto c. Left, HLE cells were stably transfected with AFP expression plasmids (AFP1# and AFP2#) or an empty vector (Vec). Right, HuH7 and HepG2 cells were transfected with an NC or siRNAs against AFP (siAFP1# and siAFP2#). (E) The expression of Bax, Bcl-2 and Cyto c in AFP-positive wild-type (*wt*) and AFP-deficient (*afp-/-*) mouse liver tumors was evaluated. (F) The expression of Bax, Bcl-2, and Cyto c in AFP-negative and AFP-positive human HCC specimens was evaluated.

Supplementary Figure 5 Overexpression of Fas suppresses HCC cell growth. (A) HLE and HepG2 cells stably transfected with Fas expression plasmids (Fas1#, Fas2#, and Fas3#) or a control vector (Vec) were established, and the protein expression of Fas was examined by Western blotting. (B) The effect of Fas overexpression on HCC cell growth was analyzed by a clonogenic assay. ***$P< 0.001$ versus Vec.

Supplementary Figure 6 AFP and Fas protein levels in HCC cell lines and tissues samples. (A) Representative immunohistochemistry (IHC) images showing AFP and Fas expression in AFP-positive wild-type (*wt*) and AFP-deficient (*afp-/-*) mouse liver tumors. *Scale bar,* 100 μm. (B) Western blot analysis of AFP and Fas expression in AFP-positive wt and *afp-/-* mouse liver tumors. (C) Western blot analysis of the expression of Fas and FasL in human HCC cells. (D) Representative IHC images showing AFP and Fas expression in AFP-negative and AFP-positive human HCC specimens. *Scale bar*, 100 μm. (E) Images showing the results for immunohistochemical staining of a tissue microarray containing 95 primary human HCC specimens.

Supplementary Figure 7 HuR inhibits Fas expression through the Fas 3'-UTR region. (A) HuR eliminated siAFP-mediated mouse Fas 3'-UTR reporter activation. Top: schematic representation of luciferase reporter constructs with the mouse Fas 3'-UTR and identified HuR-binding sequences Seq1 and Seq2. *P< 0.05 and **P< 0.01. (B-C) Overexpression of HuR suppressed human and mouse Fas 3'-UTR luciferase reporter activity. **P< 0.01 and ***P< 0.001 versus Vec. (D) Overexpression of HuR inhibited Fas protein expression in HCC cells. (E) The effect of AFP knockdown on HuR mRNA transcription was examined in HuH7 and HepG2 cells.

Supplementary Figure 8 Oxaliplatin induces cell death through the AFP-mediated apoptotic pathway. (A) The effects of several anticancer drugs on the expression of AFP and Fas in HuH7 and HepG2 cells were examined. The cells were treated with the indicated concentrations of drugs for 24 hr. (B) Oxaliplatin (Oxa) suppressed the transcription of AFP in HCC cells. The cells were treated with the indicated concentrations of oxaliplatin for 24 hr, and the mRNA level of AFP was determined by qRT-PCR. (C) Oxaliplatin (Oxa) increased the expression of Fas/FADD in HCC cells. Cells were treated with the indicated concentrations of

oxaliplatin for 24 hr. (D) The oxaliplatin-induced cell membrane Fas distribution was detected by immunofluorescence staining and confocal microscopy imaging. Scale bar, 20 μm. (E) Oxaliplatin did not activate Fas expression in AFP-negative HCC cells. The cells were treated with the indicated concentrations of oxaliplatin for 24 hr. (F) Knocking down AFP expression attenuated oxaliplatin-induced Fas/FADD activation. HepG2 cells were infected with a lentivirus containing an shRNA that interfered with AFP expression (shAFP) or a nontargeted shRNA (shNC). The cells were reseeded in a 6-well plate overnight and then incubated with the indicated concentrations of oxaliplatin for 24 hr.

Supplementary Figure 9 AFP-positive HCC cells were more sensitive to oxalipaltin-induced cell apoptosis. (A) The chemosensitivity of AFP-positive (HuH7/HepG2) and AFP-negative (HLE/SNU475) HCC cells to oxaliplatin was examined by a clonogenic assay. The cells were treated with the indicated concentration of oxaliplatin for 40 hr and allowed to grow into colonies for 12-14 days. (B) The effect of oxaliplatin on cell apoptosis induction in AFP-positive or AFP-negative HCC cells was evaluated. The cells were incubated with 40 μM oxaliplatin for 48 hr, and cell apoptosis was analyzed by flow cytometry. Apoptotic cells (%, Q2+Q4) are reported as the mean ± SD of three replicate experiments. ***P< 0.001. (C) The effects of sorafenib on the activation of Fas/FADD apoptotic signal in HuH7 and HepG2 cells. The cells were treated with the indicated concentrations of sorafenib for 24 hr.
