# Supplementary material for: AFP promotes HCC progression by suppressing the HuR-mediated Fas/FADD apoptotic pathway
Source: Cell Death Dis. 2020 Oct 2;11(10):822. doi: 10.1038/s41419-020-03030-7 (PMC7532541; doi:10.1038/s41419-020-03030-7)
Supplement: Supplementary file 11 — Supplementary Table 1 [file 41419_2020_3030_MOESM11_ESM.pdf]

Supplementary Table 1

Primers used for genotyping, gene cloning, qRT-PCR, and siRNA knockdown.

Gene	Forward primer (5'-3')	Reverse primer (5'-3')	Application
Afp	P1: CGCCGCCTGAACTACT GAAACAAT	P2: CCGGAACTAGACATGA GACCC<br>P3: GAAGAGTTCTTGCAGC TCGGT	*Afp-/-* mice genotyping
Fas	CGGAATTCGCCACCATGCT GGGCATCTGGACCCT	GCTCTAGAGACCAAGCTTT GGATTTCAT	Cloning
HuR	CGGAATTCGCCACCATGTCT AATGGTTATGAAGAC	GCTCTAGATTTGTGGGACTT GTTGGTTTTGA	Cloning
Human Fas promoter	GGGTACCAAGCTTTTTTGG CTACATTTTT	CAGATCTGGTTGTTGAGCA ATCCTCCGAA	Cloning
Human Fas 3’-UTR	CTAGCTAGCAGTGAAAAAC AACAAATT	CTAGCTAGCTGTTAAGAAAA CATGGTT	Cloning
Mouse Fas 3’-UTR	GCTCTAGAAAACTACCTCA GTTCCAGCCAT	GCTCTAGAGAAATGCAAAA AGAGATACTT	Cloning
AFP	CCAACAGGAGGCCATGCTT GCTT	GAATGCAGGAGGGACATAT GTTT	qRT-PCR
Fas	TGGGGTGGCTTTGTCTTCTT	TCTGTTCTGCTGTGTCTTGG A	qRT-PCR
HuR	CACAGCTTGGGCTACGGCT TTGTG	AGGACCCGCGAGTTGATGA TCCG	qRT-PCR
GAPDH	ACGGATTTGGTCGTATTGGG C	CTCGCTCCTGGAAGATGGT GAT	qRT-PCR
siAFP1#	AUAAGUGUCCGAUAAUAAUGUCAGC		Gene silence
siAFP2#	CCAGAACACUGCAUAGAAATT		Gene silence

shAFP	AUAAGUGUCCGAUAAUAAUGUCAGC	Gene silence
Nontarget siRNA (, NC)	UGGUUUACAUGUCGACUAA	Gene silence
